# Performance of a scalable RNA extraction-free transcriptome profiling method for adherent cultured human cells

**DOI:** 10.1038/s41598-021-98912-x

**Published:** 2021-09-30

**Authors:** Shreya Ghimire, Carley G. Stewart, Andrew L. Thurman, Alejandro A. Pezzulo

**Affiliations:** Department of Internal Medicine, Roy J. and Lucille A. Carver College of Medicine, University of Iowa, Iowa City, IA USA

**Keywords:** Quality control, High-throughput screening, Transcriptomics, Bioinformatics, Gene expression analysis, Genomic analysis, High-throughput screening, Isolation, separation and purification

## Abstract

RNA sequencing enables high-content/high-complexity measurements in small molecule screens. Whereas the costs of DNA sequencing and RNA-seq library preparation have decreased consistently, RNA extraction remains a significant bottleneck to scalability. We evaluate the performance of a bulk RNA-seq library prep protocol optimized for analysis of many samples of adherent cultured cells in parallel. We combined a low-cost direct lysis buffer compatible with cDNA synthesis (in-lysate cDNA synthesis) with Smart-3SEQ and examine the effects of calmidazolium and fludrocortisone-induced perturbation of primary human dermal fibroblasts. We compared this method to normalized purified RNA inputs from matching samples followed by Smart-3SEQ or Illumina TruSeq library prep. Our results show the minimal effect of RNA loading normalization on data quality, measurement of gene expression patterns, and generation of differentially expressed gene lists. We found that in-lysate cDNA synthesis combined with Smart-3SEQ RNA-seq library prep generated high-quality data with similar ranked DEG lists when compared to library prep with extracted RNA or with Illumina TruSeq. Our data show that small molecule screens or experiments based on many perturbations quantified with RNA-seq are feasible at low reagent and time costs.

## Introduction

RNA sequencing (RNA-seq) is a popular tool in modern biology that has expanded our understanding of the regulation and complexity of the transcriptome of many species. Primarily used for differential gene expression analysis, other applications include transcript variants detection, alternative splicing, pathway analysis and exploring cellular heterogeneity and diversity in stem cell biology^1,2^. The typical RNA-seq workflow starts with RNA extraction from samples, followed by cDNA synthesis, adaptor ligation, size selection and high-throughput sequencing. The sequences are aligned to a reference genome and/or transcriptome to generate a genome wide transcription map which includes the transcriptional structure and/or expression profile for each gene^3^.

RNA-seq workflows have become progressively more efficient over time. There are at least 100 distinct RNA-seq library prep protocols^2^. By studying the effects of many perturbations (alterations induced by treatments) in parallel on cellular gene expression, efforts such as the Connectivity Map^4^ have uncovered novel mechanisms of disease and therapeutic targets. However, significant bottlenecks impede adoption of highly parallelized methods in the absence of robotics equipment. Highly efficient RNA extraction is simple to perform in a small number of samples but becomes costly and laborious at the scale of hundreds of samples, particularly for adherent cells that may require dissociation prior to processing. Moreover, highly multiplexed RNA-seq library prep generally requires costly commercial kits.

Prior work has demonstrated that qPCR can be accurately performed using bulk human primary and transformed/cancer cell samples without RNA extraction. These methods are based on the use of a lysis buffer compatible with in-lysate cDNA synthesis and downstream sample processing and have been applied to single cell RNA-seq^5–9^. Recently, Foley et al.^10^ developed a simple method (Smart-3SEQ) for rapid RNA-seq library prep; this was tested with laser micro dissected tissue applied to cDNA synthesis and with purified RNA, taking up to 4 h on average and with low reagent costs.

We hypothesized that in-lysate RNA-seq library prep with Smart-3SEQ would reduce reagent and time costs while maintaining quality comparable to a gold standard. We did side-by-side comparisons of library prep in-lysate vs. using purified RNA, followed by either Smart-3SEQ or Illumina TruSeq. We found that in-lysate cDNA synthesis allows generation of high-quality sequencing data without RNA input normalization. The gene expression profile of in-lysate RNA highly correlated with purified RNA. When compared to our gold standard, both methods showed similar performance for detection of DEGs and for pathway analysis. Thus, our data suggests that in-lysate RNA-seq library prep with Smart-3SEQ facilitates high-quality RNA-seq analysis of cultured adherent cells.

## Results

### Experimental design overview

**Figure 1:**
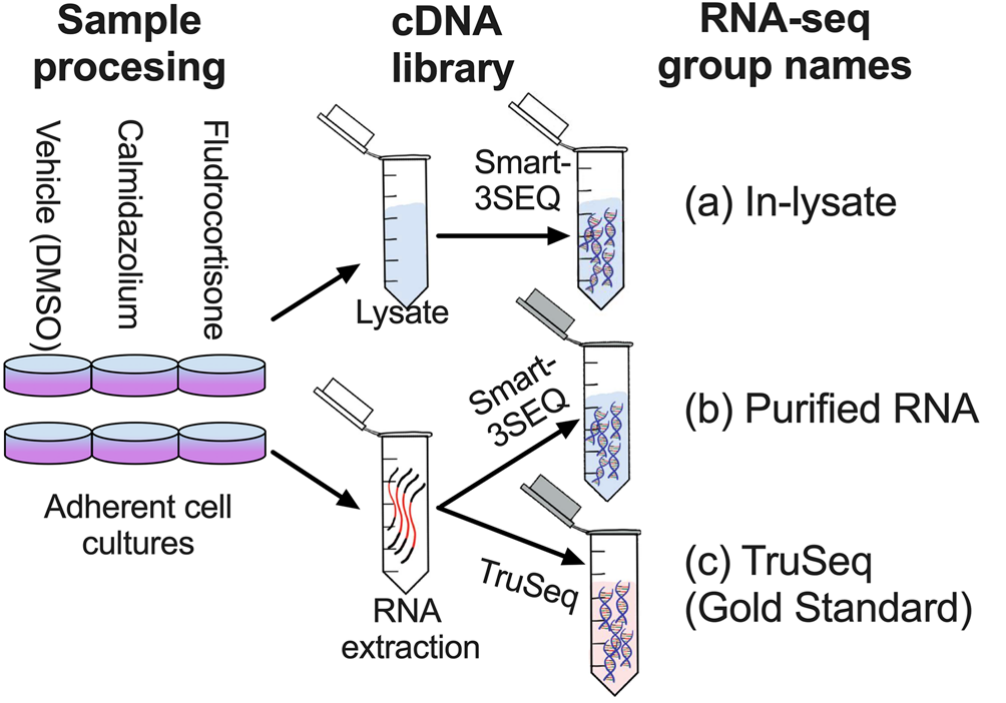
**In-lysate RNA seq library prep performance: experimental overview.** We used Smart-3SEQ to compare RNA-seq libraries prepared directly from lysate (**a**) and from extracted RNA (**b**). We used TruSeq libraries from matching RNA as the gold standard (**c**). 18 samples (6 donors, 3 treatments) were used for each method.

We performed an experiment comparing RNA-seq library prep using cDNA synthesis with in-lysate RNA versus RNA purified from the same samples. We chose to use Smart-3SEQ library prep because it has fewer steps and uses lower volume of common reagents compared to many other RNA-seq library prep methods. It allows accurate quantification of transcript abundance with low RNA input making this approach rapid, robust and cost-efficient^10^. We further used purified RNA for TruSeq RNA-seq library prep as a gold standard (Fig. 1). Samples consisted of human dermal fibroblasts from 6 different human donors exposed to DMSO (Vehicle,10 μM), calmidazolium (10 μM), or fludrocortisone (10 μM) for 6 h. These drugs were chosen as they are known to affect multiple genes in pathways active in dermal fibroblasts. Cell culture replicates were then used for direct lysis vs. silica column-based RNA extraction. We then quantified RNA concentration in purified RNA samples using various methods (Supplementary Table S1). 1 μL of sample or 5 ng RNA in 1μL was used for in-lysate and purified RNA Smart-3SEQ library prep, respectively. 500 ng purified RNA per sample was used as input for preparing TruSeq stranded mRNA libraries following standard Illumina protocols. Fragment size distribution of pooled libraries were measured using High Sensitivity DNA Assay (Supplementary Fig. S1). We then used various analysis packages as described in the Methods section to generate uniquely aligned gene count tables. Supplementary Table S2 shows the percentage of total sequenced reads that were aligned to the reference genome by HISAT2 v2.1.0. A summary of FeatureCounts run is provided in Supplementary Table S3.

### Effects of variable RNA mass loading on in-lysate RNA-seq library prep

**Figure 2:**
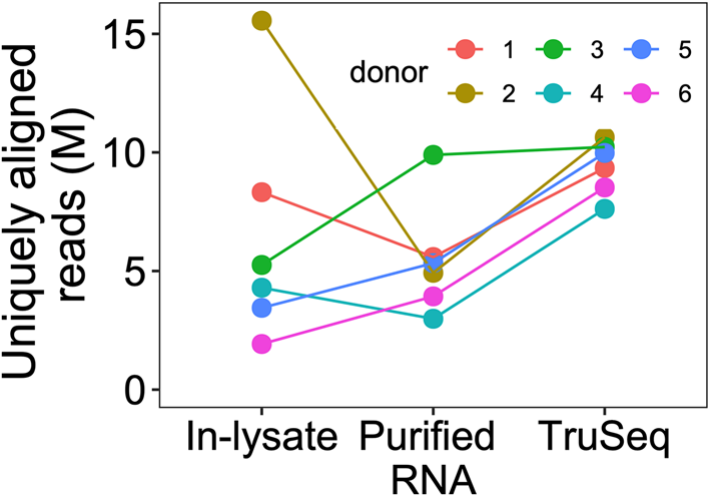
**In-lysate Smart-3SEQ RNA-seq library prep results in similar aligned sequence counts as TruSeq.** Each dot represents a donor for a vehicle-treated sample. The line connects same donor sample among methods. The y-axis shows total uniquely aligned gene reads by featureCounts in millions (M).

Normalization (equalization of concentration and volume) of RNA input at scale is costly and laborious, so we examined the performance of normalization free in-lysate cDNA synthesis. For in-lysate samples, RNA input was neither normalized for cDNA library prep nor for sequencing. For purified RNA samples and for TruSeq (gold standard) samples, RNA input was normalized for cDNA library prep based on Qubit RNA quantification. Figure 2 shows the distribution of total uniquely aligned gene reads for DMSO treated samples across three RNA-prep methods. As expected, the total counts of normalized TruSeq libraries were more uniform than those of libraries prepared without RNA input normalization. Gene counts for calmidazolium and fludrocortisone treated samples showed larger variability than those of vehicle-treated samples (Supplementary Fig. S2). These data suggest that in-lysate cDNA synthesis allows RNA-seq library prep without RNA input normalization. We then investigated the effects of library prep methods on RNA-seq gene expression quantification accuracy.

### Gene expression profiles of in-lysate and purified RNA library prep highly correlate

**Figure 3:**
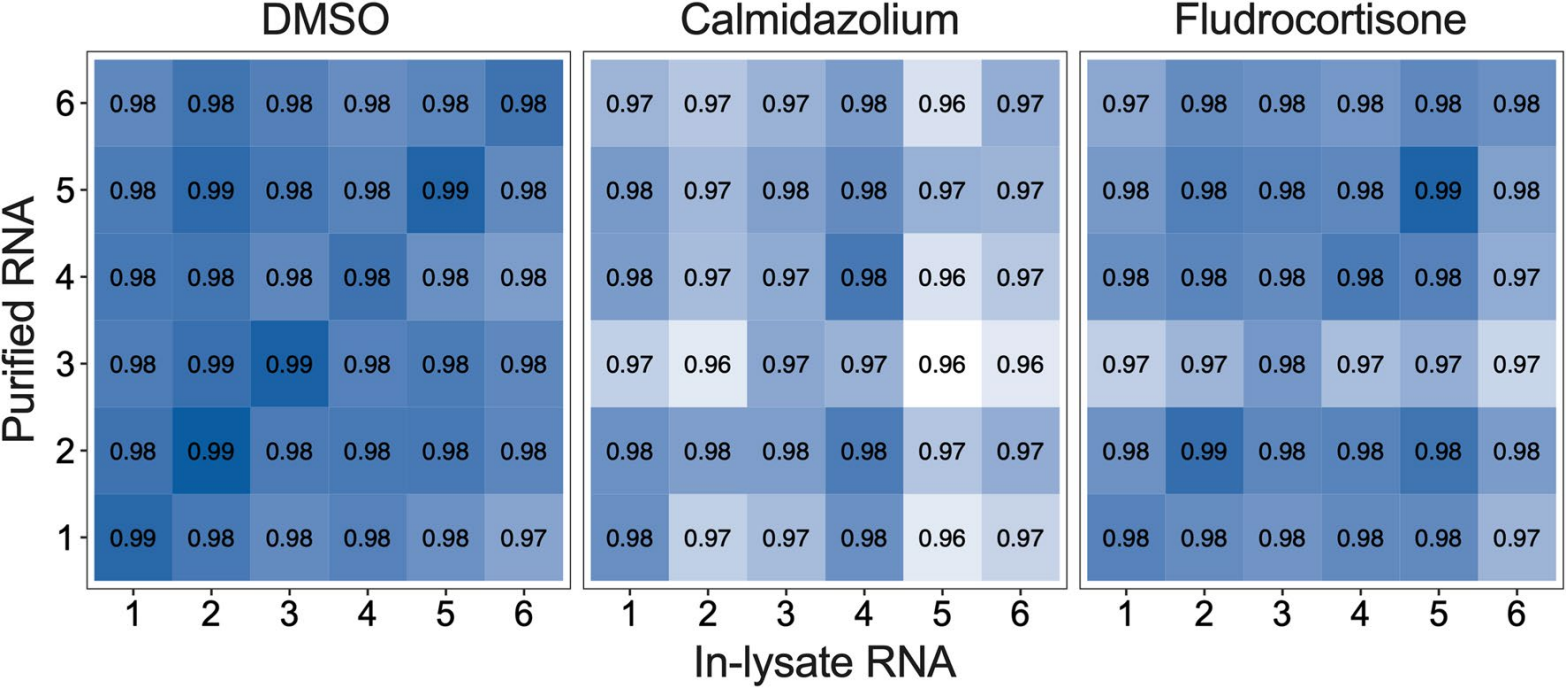
**In-lysate and Purified RNA Smart-3SEQ RNA-seq data highly correlate.** Gene counts correlation between data from RNA-seq libraries prepared from purified and in-lysate for DMSO-, calmidazolium-, and fludrocortisone-treated samples. Values in each plot represents R^2^ value for Pearson's correlation. Uniquely aligned gene reads were log2-transformed prior to calculating the correlation.

Use of in-lysate RNA for RNA-seq library prep may result in unexpected biases in the type (nuclear vs. cytoplasmic, length) of mRNA molecules recovered that could either mask or accentuate variation between samples. We hypothesized that in-lysate and purified RNA library prep for RNA-seq would result in highly correlated data from matching samples. We transformed the uniquely aligned gene reads into log2 counts per million (CPM) and performed Pearson’s correlation within each treatment group: DMSO (vehicle), calmidazolium and fludrocortisone (Fig. 3). Out of three treatment groups, DMSO showed highest correlation between methods, followed by Fludrocortisone and Calmidazolium. Overall, samples correlated more highly with themselves than with other samples across methods. These data show that gene expression profiles generated from RNA-seq libraries prepared with in-lysate versus purified RNA are highly comparable. We further performed Pearson’s correlation between Smart-3SEQ libraries (in-lysate and Purified RNA) and TruSeq for each treatment group (Supplementary Fig. S3). Additional gene length-based normalization was done for TruSeq data (uniquely aligned gene reads/gene length) prior to conversion to log2cpm, in order to perform direct comparison with Smart-3SEQ libraries. Both Smart-3SEQ libraries showed similar correlation to TruSeq ranging from 0.93 to 0.96 for matching samples.

### In-lysate and purified RNA Smart-3SEQ library DEG analysis show response patterns similar to TruSeq

**Figure 4:**
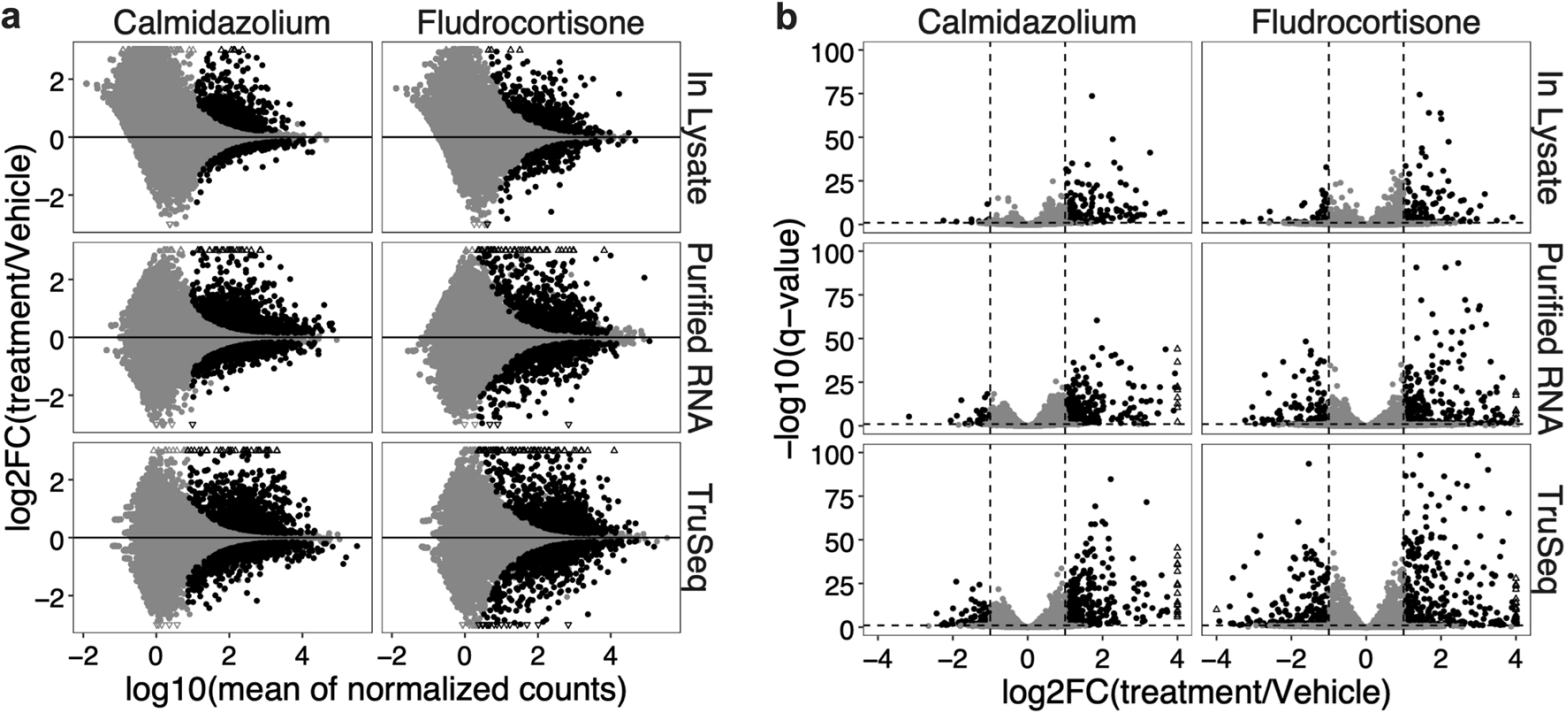
**DEG analysis of RNA-seq data from purified, in-lysate and TruSeq RNA-seq libraries.** (**a**) MA plots, genes with q-value < 0.1 are labeled black. (**b**) Volcano plots (cutoffs log2FC + / − 1 and q-value 0.1) for calmidazolium- vs fludrocortisone-treated dermal fibroblast RNA-seq DGE analysis. Triangles in plots indicate genes outside of the plotting window.

Variable sequencing depth can affect quantification of low-expressed genes for accurate differential expression analysis. Since the gene counts in matching samples between methods highly correlated with each other (Fig. 3) we hypothesized that our data would produce similar number of significant DEGs between methods for each treatment group. We performed DEG analysis (DESeq2-1.30.1^11^, design: ~ donor + treatment) analysis for each method using uniquely aligned gene reads. Figure 4a shows MA plots for each method and treatment groups. DESeq2 uses the average expression strength of each gene across all samples as its filter and then removes all genes with mean normalized counts below a certain threshold, calculated using multiple tests. The chosen threshold, by default maximizes the number of genes found at a specified target FDR (0.01). In Fig. 4a, the genes that contain enough information to yield a significant call at an estimated FDR of 0.01 are colored black. Supplementary Table S4 summarizes the number of DEGs, non-DEGs and filtered genes identified for each method and comparison groups. The calmidazolium treatment seems to induce more changes in gene expression compared to fludrocortisone treatment group for each method. The in-lysate method had the lowest number of DEGs for Fludrocortisone treatment group compared to Purified and TruSeq method. As expected, TruSeq method identified more DEGs for both treatment groups. We then compared the significant genes using specific thresholds for log2 fold change (LFC) and q-value. Figure 4b shows volcano plots in which, using cutoffs of abs(log2FC) = 1 and q-value < 0.1, both Calmidazolium and Fludrocortisone induced comparatively more upregulated than downregulated genes in all three methods. Supplementary Table S5 summarizes the number of significant and non-significant genes (up and downregulated) for each method and comparison groups. Purified RNA samples had more significant genes in both treatment groups compared to in-lysate RNA samples. TruSeq samples had the highest number of identified significant genes as expected. Overall, both Smart-3SEQ methods reflect similar differential gene expression patterns however, the Purified RNA method had more similarity to TruSeq compared to in-lysate method. Next, we examined the magnitude correlation of differentially expressed genes between two methods.

### The effect sizes of significant DEGs from in-lysate and purified RNA highly correlate with each other

**Figure 5:**
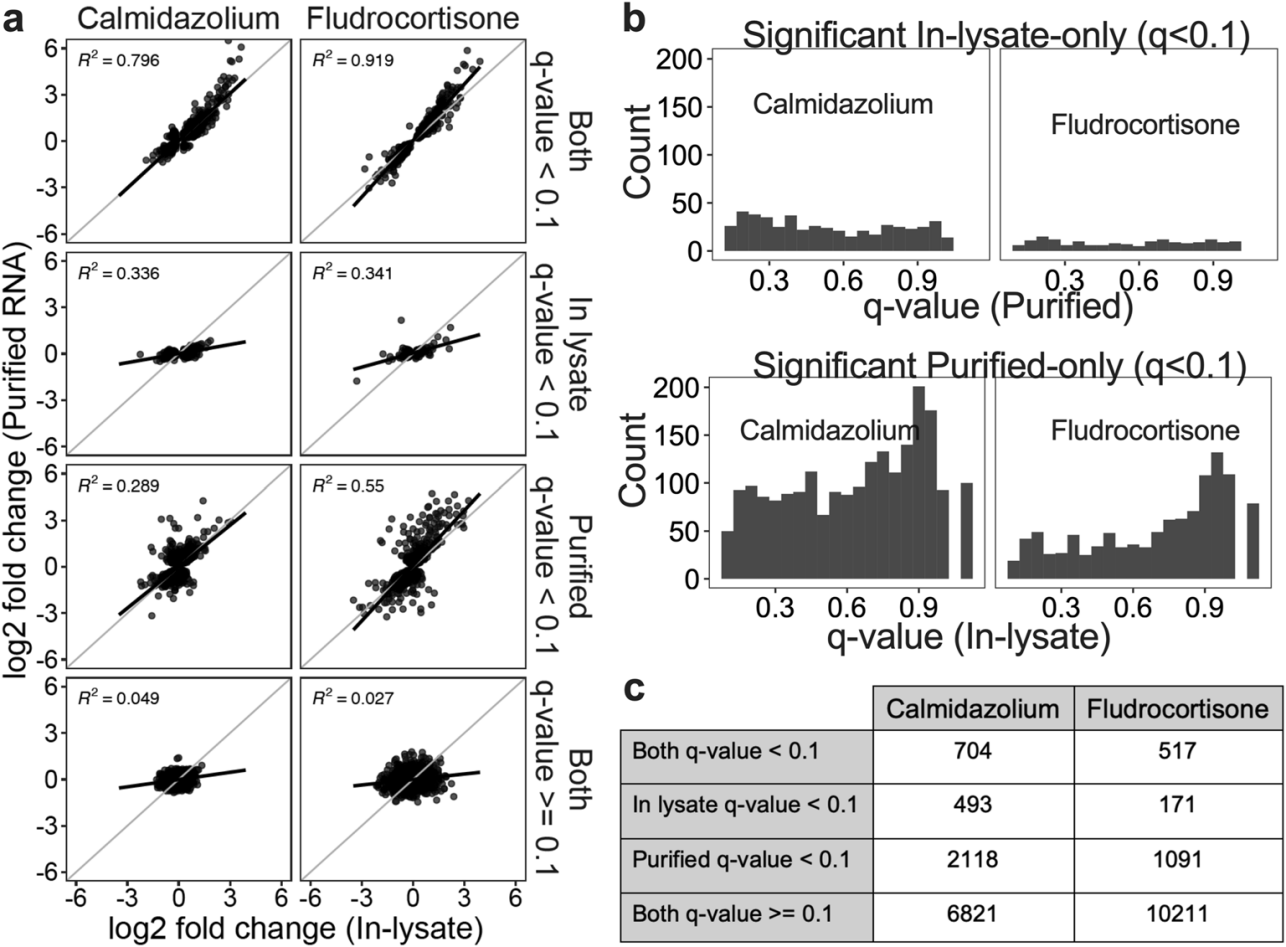
**The magnitude of differential gene expression measured with In-lysate and Purified RNA Smart-3SEQ RNA-seq library prep highly correlates.** (**a**) F–F plot: log2 fold change of Purified vs In-lysate RNA for genes with specified q-value (labeled on right) (**b**) q-value distribution of genes. Plots show distribution of non-significant q-values for corresponding method in which the q-value was < 0.1. Gap near value of 1 is due to only non-detected genes having a q-value = 1. (**c**) Summary of significant vs. non-significant genes for each method/group.

Since both MA and Volcano plots in Fig. 4, showed similar differential gene expression patterns between Purified and in-lysate method, we hypothesized that the effect size (log fold change—LFC) of DEGs from purified and in-lysate RNA samples would highly correlate with each other. Using the LFC and q-values, we generated “F-F plots” to further examine the relationship between DEGs from the different methods tested. As shown in Fig. 5a, purified and in-lysate RNA genes showed positive linear correlation for genes with q-value < 0.1 in both methods. R^2^ for these genes between methods was 0.796 and 0.919 for calmidazolium and fludrocortisone treatment groups, respectively. The data show high positive correlation between the LFC of genes that are expressed in high levels and considered significant in both methods. For genes significant in one method only (In-lysate- or Purified-only), we analyzed the distribution of q-values in the corresponding non-significant method (Fig. 5b). We found that non-significant q-values were evenly spread between 0.1 and 1. This suggests that DEG calling did not depend solely on systematic effects due to sample size or gene variability. Figure 5c summarizes number of genes in each treatment groups and methods with q-value cutoff. Next, we examined this concordance with respect to our gold standard (TruSeq) using rank-based and binary classification methods.

### In-lysate and purified RNA show similar performance for calling DEGs

**Figure 6:**
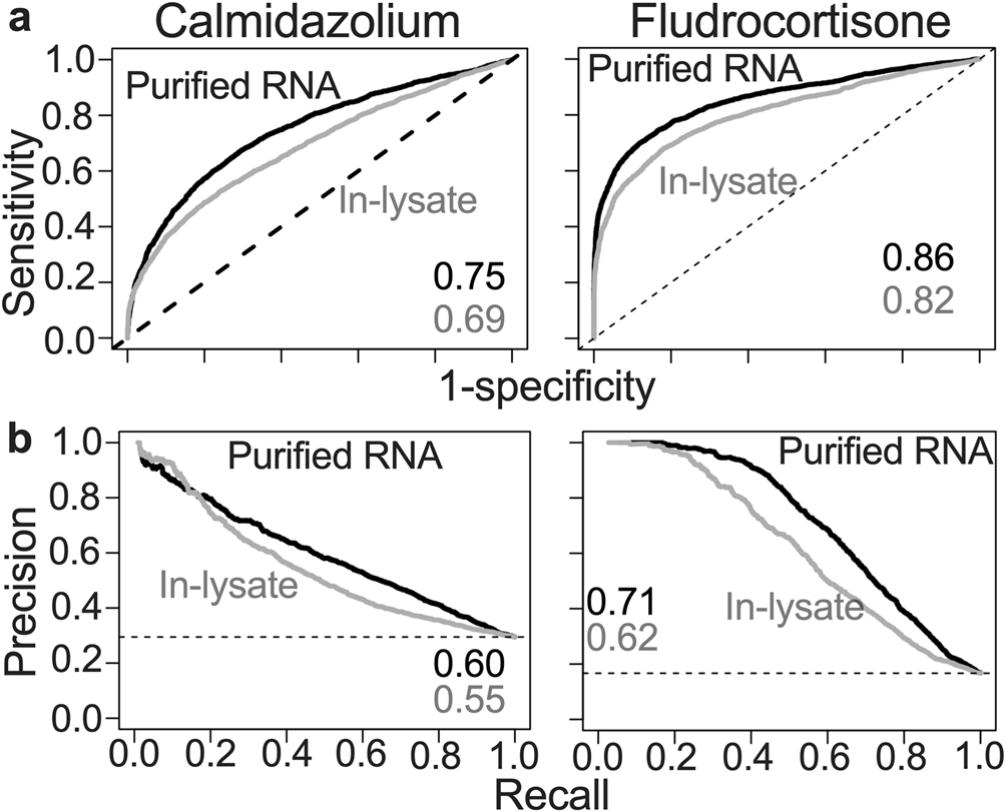
**In-lysate and purified RNA Smart-3SEQ library prep accurately detect DEGs compared to TruSeq.** Differentially expressed genes list for calmidazolium and fludrocortisone treated dermal fibroblast from in lysate and purified RNA were compared to Illumina TruSeq as a gold standard. True positives were identified as those genes in Smart3-SEQ methods with FDR < 0.1 and abs(log2FC) > 1. (**a**) Receiver Operating Curve (ROC) and (**b**) Precision-Recall curves (PR) were measured. Area under the curve (AUC) for each performance measurement is indicated in the figure.

To further investigate how the gene expression pattern from in-lysate RNA and purified RNA library preps compare in performance, we plotted Receiver Operating Characteristic (ROC) and Precision-Recall (PR) curves for each treatment group (Fig. 6a, b, respectively), using the TruSeq dataset as a gold standard. We included PR curves in addition to ROC because of the high imbalance in our data for the positive (DEGs) and negative (non-DEGs) classes (Supplementary Table S4). To produce these curves, we used ROCR-1.0-11 package with different cutoffs for q-values between zero and one, and the “true” class labels were those genes with q-value < 0.1 for TruSeq. Since Purified RNA method showed greater similarity to TruSeq compared to in-lysate method (Fig. 4), we hypothesized that purified RNA would perform better than in-lysate RNA when compared to our gold standard. ROC and PR curves showed that both methods performed better for the fludrocortisone treatment group compared to calmidazolium (Fig. 6). Overall, purified RNA libraries performed slightly better than in-lysate RNA libraries for each treatment group and classification method. These data suggest that in-lysate RNA-seq library prep has a slight negative effect on performance of DEG analysis.

### Similar DEG sets are called with in-lysate and purified RNA-seq library prep

**Figure 7:**
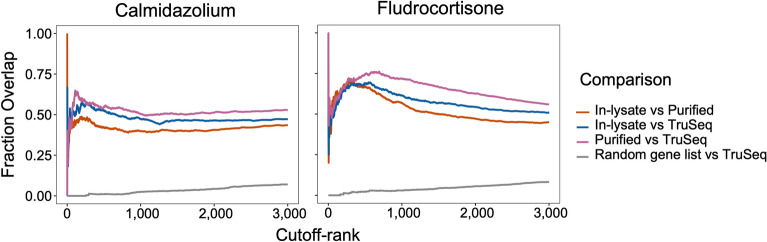
**In-lysate and Purified RNA Smart-3SEQ library prep DGE analysis ranks genes similarly to TruSeq.** DGE lists were sorted by q-value and abs(log2FC). For each rank position, the % overlap between lists is shown. A randomly re-ranked list for all detected genes was compared to the TruSeq data. Figure shows top 3000 gene ranks for each method.

One of the main goals of DEG analysis is to prioritize lists of genes highly likely to be differentially expressed between two conditions for subsequent pathway analysis. DEG lists generated with arbitrary FC and q-value cutoffs can be affected by methods used for RNA-seq library prep or data analysis, variable depths or reference transcriptomes. In contrast, rank-based methods allow easier comparisons between lists by focusing on genes/transcripts enriched near the top of the rankings^12–16^. We hypothesized that data from in-lysate RNA and purified RNA library preps ranked by q-value and absolute fold change would highly correlate near the top of the ranked list when compared to data from TruSeq libraries as a gold standard. We found that there was a high (50–75%) overlap in lists of DEGs when the threshold used was between 300 and 600 top DEGs (Fig. 7). This data suggests that between 300 and 600 genes are likely to be modulated by calmidazolium or fludrocortisone in human dermal fibroblasts. Moreover, these data suggest that both methods produce ranked lists that are highly comparable to our gold standard for RNA-seq library prep, and would yield similar results in downstream functional analysis of DEG lists.

### Pathway analysis reflects high agreement among top enriched pathways across methods

**Figure 8:**
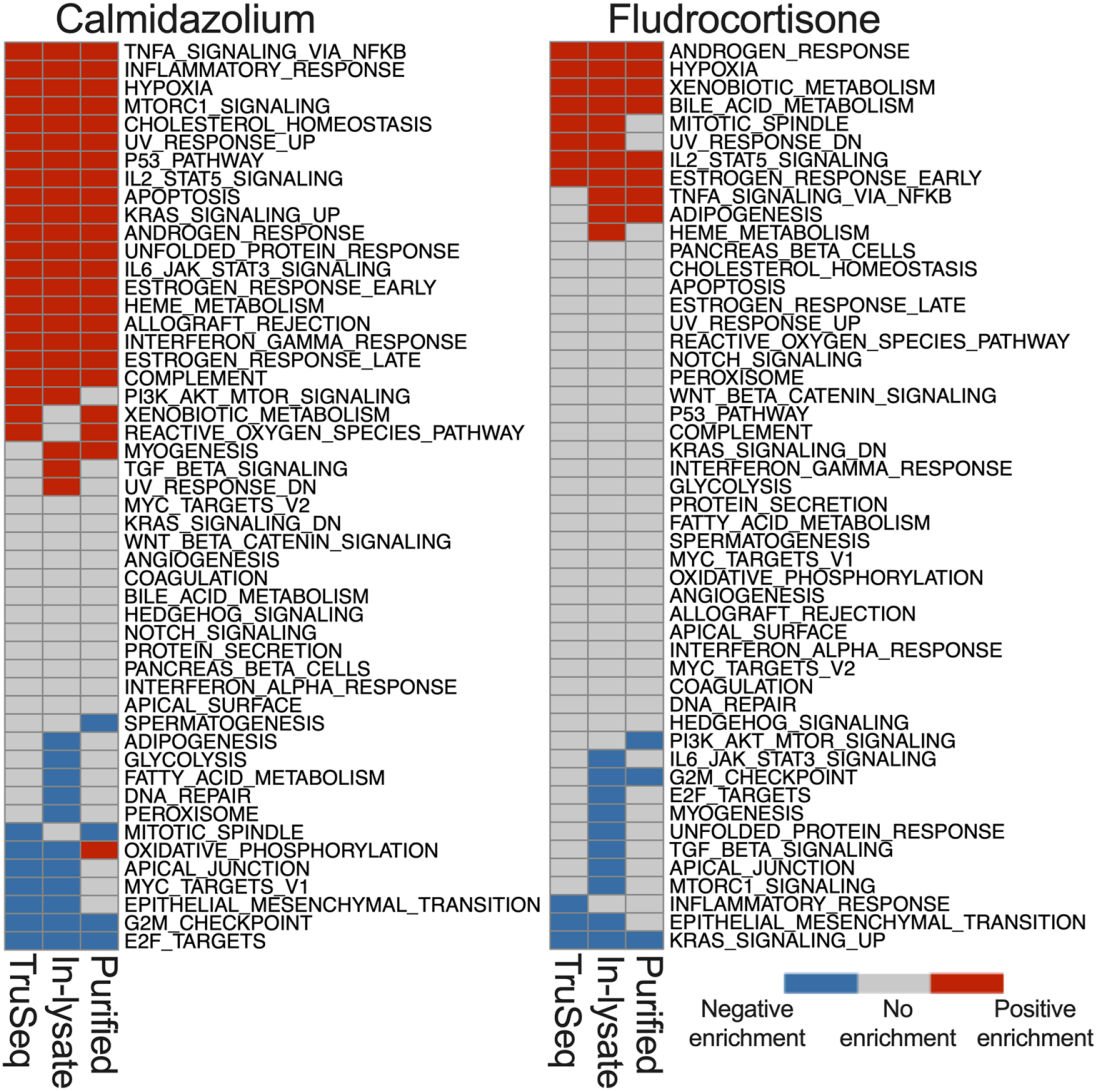
**In-lysate and Purified RNA Smart-3SEQ library RNA-seq detects similar enriched pathway as TruSeq.** Gene set enrichment analysis performed using fgsea and MSigDB Hallmark gene set. Heatmaps shows pathway enrichment among methods. Legend: Positive enrichment refers to the pathways with enrichment score > 1, q-value < 0.05, No enrichment: q-value ≥ 0.05, negative enrichment: enrichment score < 1, q-value < 0.05.

We performed gene set enrichment analysis using the fgsea package^17^ and hallmark gene set from MSigDB^18^. We chose the hallmark gene set as it summarizes and represents specific well-defined biological states or processes^18^. We then ranked the genes using Wald-statistic values from DESeq2 results for each method^11^. Figure 8 summarizes the pathway enrichment for each comparison groups. Pathways with enrichment q-value > 0.05 were categorized as “no enrichment”, while pathways with enrichment q-value < 0.05 were categorized as either positive or negative enrichment based on their enrichment score > 1 or < − 1, respectively.

Overall, there is high agreement across methods for pathways detected. This suggests that in-lysate Smart-3SEQ RNA-seq library prep from adherent cultured cells accurately identifies enriched pathways.

## Discussion

Recent methods for transcriptome analysis on single cells show that RNA extraction can be skipped by using lysis buffers compatible with cDNA synthesis. These studies suggest that direct lysis results in adequate RNA recovery compared to column or magnetic beads-based RNA extraction methods which makes direct lysis rapid, cheap and highly effective^5^. Here, we expand this concept to RNA-seq library prep using samples of adherent cells growing in multi-well plates, which are a common high throughput assay model.

We demonstrate that high quality bulk RNA-seq can be performed without RNA isolation for library prep. Moreover, when combined with methods for simplified multiplex library prep using off the shelf reagents, significant time and reagent cost savings are achieved. Performing in-lysate Smart-3SEQ library prep, we have shown the success of our approach: (1) all quality control criteria were achieved, and (2) gene response profiles and pathway analysis reported by in-lysate and purified RNA library highly correlate with each other. Thus, the approach presented here enables rapid, robust and highly cost-efficient RNA-seq of adherent cell samples in parallel. The reagent costs for generating lysates used for library prep are negligible compared to RNA extraction using silica columns or magnetic beads. Moreover, this approach takes only approximately 4–6 h from sample processing to final sequencing-ready library (Supplementary Table S6).

However, there are some limitations to the approach presented here. The amount of RNA loaded for in-lysate cDNA synthesis was not normalized which resulted in variable and low-sequencing depth across samples (Supplementary Table S3). Although this issue didn’t negatively affect the quality or the gene expression profile of the samples, we did detect lower numbers of DEGs from in-lysate library prep. Moreover, Smart-3SEQ sequences only the 3′ fragment with Poly(A) tail of each transcript. Although this allows for a simple and accurate quantification of transcript abundance, information regarding splicing or genotypes cannot be detected unless the splicing junction is near the 3′ end of the transcript^10^. We used a matching dataset generated with libraries prepared from extracted RNA and processed with the Illumina TruSeq as a gold standard. Whereas a digital gene expression method such as Smart-3SEQ is not expected to match the data generated with a full-length transcript library prep method like TruSeq, it is an appropriate gold-standard as it’s the most frequently used library prep protocol. We detected a decrease in within-sample correlation between methods upon small-molecule perturbation compared to vehicle-treated samples. This effect was more marked with calmidazolium treatment. We speculate that: (1) the expression profile of human fibroblasts may become more similar between individuals upon stimulation, (2) since the lysis buffer leaves nuclei intact^5–9^, in-lysate RNA library prep captures mRNA exported to the cytoplasm which may occur differentially between individuals, or (3) perturbation with calmidazolium may cause changes undetected by our methods in the ability to correctly fragment and process mRNA.

Other transcriptional profiling platforms have been developed that facilitate processing of hundreds of samples in parallel. Luminex L1000, used for the Connectivity Map^4^ is a cost-effective platform for transcriptional profiling; however, it only allows the measurement of approximately 1000 genes making this approach less efficient and more susceptible to signal–noise problems^19^. Moreover, the inference method applied to estimate expression of the remaining transcriptome may not be accurate for specific sample types. Pooled library amplification for transcriptome expression (PLATE-Seq) is another low-cost approach for genome wide profiling which highly compares with other large-scale profiling, however it is based on RNA purification^4^. Another approach, Digital RNA with perturbation of Genes (DRUG-seq) skips RNA extraction by using the direct lysis approach and cuts down the library prep time and cost. Here, we chose SMART-3SEQ as it relies on chemical fragmentation of RNA rather than tagmentation of pooled libraries which is costly and can be variable. The approach presented here is therefore highly compatible with high throughput drug screening at a low cost.

Highly-parallel assessment of perturbation-transcriptome responses in biological samples can be an unbiased and data-rich approach to screen compounds and to test hypothesized mechanisms of disease. Other novel approaches such as single cell RNA-seq-based genetic perturbation analysis together with small-molecule-based approaches as presented here provide a nimble toolbox to uncover novel biology.

## Methods

### Cell culture

Human primary dermal fibroblasts (see below for ethics declaration and IRB approval) were obtained using the method described in^20^. Cells were maintained in DMEM (Gibco, Dublin, Ireland, Item No. 11965-092) with 20% FBS (Gibco, Dublin, Ireland, Item No. 26140-079) and Pen/Strep 1:100 (Gibco, Dublin, Ireland, Item No. 15140-122) and gentamycin 25 μg/mL (IBI Scientific, Dubuque, IA, Item No. IB02030), at 37 °C with 5% CO_2_ and were used at passage #4, near confluency, in 24 well plates. Samples were treated for 6 h as indicated with 10 μM Calmidazolium chloride (Cayman Chemical Company, Ann Arbor, MI, Item No. 14442) or Fludrocortisone acetate (Sigma-Aldrich, St. Louis, MO, Item No. F6127) or DMSO (10 μM).

### RNA preparation

Total RNA was directly extracted and purified from cell cultures using RNeasy plus mini kit from QIAGEN (Hilden, Germany). In-lysate RNA was prepared by washing cell cultures with PBS and incubating them in lysis buffer for 5 min while on ice. The lysis buffer was adapted from 5 to 9 and consists of 0.1% (1 mg/mL) BSA (New England Biolabs, Ipswich, MA, Item No. B9000S) and 0.3% Igepal CA 630 (Sigma-Aldrich, St. Louis, MO, Item No. I8896. Based on these studies, we estimated an appropriate cell/buffer volume ratio to be between 100 and 500 cells per μL buffer for adherent cells, as an excessively high ratio may negative affect compatibility with cDNA synthesis. For our cell culture vessel (24 well plate), near confluent cultures correspond to an average of 100,000 cells/well. We chose a ratio of 200 cells/μL buffer, so 500ul of Igepal/BSA buffer was used per well. The lysate was aspirated and was only mixed with the pipette tip once in a collection tube. Total RNA concentrations were measured using Qubit HS RNA, Trinean DropSense 16 and Nanodrop (Thermo Fischer Scientific, Waltham, MA) and, RNA quality was measured by Agilent BioAnalyzer (Supplementary Table S1). Samples were stored at − 80 °C until library prep.

### Library preparation

Extracted RNA and cell lysate (above) was used for preparing sequencing libraries according to the Smart-3SEQ protocol v1.9^10^. Smart-3SEQ multiplexing was done by pooling equal sample volumes in the Pre-SPRI pooling option, with the universal P5 and indexed P7 primer scheme. Illumina TruSeq libraries were prepared at the University of Iowa Genomics Facility using standard Illumina TruSeq stranded mRNA protocols. Concentration and fragment size of prepared library was measured using Qubit HS and High Sensitivity DNA Assay. Fragment size for Smart-3SEQ library was ~ 500 bp which falls within the recommended 200–600 bp (Supplementary Fig. S1).

### Sequencing

3 ng/ul of pooled cDNA library was used as sequencing input for each Smart-3SEQ libraries. Smart-3SEQ cDNA libraries were prepared with a 40% spike-in of phiX and sequenced on Illumina NovaSeq 6000 SP Flowcell instrument (approximately 400M PE Reads per lane) configured for 100nt read 1 and 8nt for the P7 index read. TruSeq mRNA-seq libraries were sequenced on two lanes of an Illumina HiSeq 4000 in 75PE configuration. 40% PhiX was added out of an abundance of caution during method implementation. This is due to our use of the two-color Illumina Novaseq 6000 system, which may generate errors when sequencing SMART-3SEQ libraries due to a trinucleotide G repeat in the second strand template-switching reverse transcription primer. We did not systematically investigate lower amounts of PhiX, which would further decrease sequencing costs.

### Data processing

The base calls were demultiplexed and converted to FASTQ format by sequencing core facility. For Smart-3SEQ data, adapters were trimmed, UMI sequence and G-overhang were extracted and polyA tails were removed according to the suggested pipeline in data processing protocol^10^. Quality control analysis was done with FastQC and MultiQC^21^. The processed fastq files were aligned using HISAT2-2.1.0^22^ and human reference genome-build GRCh38.p13 (accession: GCF_000001405.39, annotation-date 12/05/2019, annotation-source NCBI Homo sapiens Updated Annotation Release 109.20191205). Uniquely aligned reads were counted by featureCounts from the Subread package-1.6.4^23^. Overall alignment rate and featureCounts summary can be found in Supplementary Tables S2 and S3. For performing pairwise correlation (Fig. 3 and Supplementary Fig. S3), uniquely aligned gene reads were transformed into log2 counts per million (CPM). For TruSeq, each gene reads were divided by the corresponding gene length before converting it to log2cpm.

### Gene expression analysis

Uniquely aligned gene reads were then used as input for differential gene expression analysis with DESeq2-1.3-0.1^11^. Pearson’s Pairwise correlation and F-F plot was created in R using various packages and functions. ROC and PR curve were plotted using ROCR-1.0-11 package^24^. For additional software packages versions and parameters, please see Supplementary Table S7. DESeq2 results are available in Supplementary Table S8.

### Ethics declaration and human subjects research approval

Human dermal fibroblast samples were obtained in compliance with the guidelines of the Declaration of Helsinki as revised in 2013. Approval was obtained from the University of Iowa Institutional Review Board, IRB# 201311783. Informed consent was obtained from the participants of the study.

## Supplementary Information

Supplementary Information 1.

Supplementary Table S3.

Supplementary Table S8.

## Data Availability

The data used in this study is available in the NCBI Gene Expression Omnibus, accession GSE164650.

## Abbreviations

DEG: Differentially expressed gene
ROC: Receiver operating characteristic
PR: Precision recall
AUC: Area under the curve
