# Supplementary material for: Performance of a scalable RNA extraction-free transcriptome profiling method for adherent cultured human cells
Source: Sci Rep. 2021 Sep 30;11:19438. doi: 10.1038/s41598-021-98912-x (PMC8484438; doi:10.1038/s41598-021-98912-x)

Performance of a scalable RNA extraction-free transcriptome profiling method for adherent cultured human cells

Shreya Ghimire[1], Carley G. Stewart[1], Andrew L. Thurman[1], Alejandro A. Pezzulo[1*]

[1]Department of Internal Medicine, Roy J. and Lucille A. Carver College of Medicine, University of Iowa, Iowa City, IA

[*]Address correspondence to:

Alejandro A. Pezzulo

University of Iowa

Email: alejandro-pezzulo@uiowa.edu

Phone: (319)335-2213

Supplementary Table S1: RNA QC values. Final elution concentrations and quality of purified RNA samples. 260/280, 260/230,28S/18S ratios and RNA quality number (RQN) indicates the quality of total purified RNA. ~2 for 260/280 and 28S/18S, RQN of 10 indicates high quality RNA samples.

Sample name	Nanodrop			Qubit HS	Fragment Analyzer		
	ng/ul	260/280	260/230	ng/ul	Conc (ng/ul)	RQN	28S/18S
samp1_DMSO	95.7	2.08	0.26	95	108.91	10	2
samp1_calmidazolium	58.4	2.07	0.79	80	81.35	10	2
samp1_fludrocortisone	93.5	2.11	0.77	96	121.89	10	2.3
samp2_DMSO	84.4	2.11	0.39	112	141.29	10	1.9
samp2_calmidazolium	54.7	2.11	0.21	81.2	74.36	10	2.2
samp2_fludrocortisone	48.8	2.14	0.76	78.6	61.34	10	2.2
samp3_DMSO	47.7	2.09	0.61	43.8	67.63	10	2.3
samp3_calmidazolium	27.7	2.03	0.19	19.1	30.34	10	2.6
samp3_fludrocortisone	44.4	2.09	0.89	36	45.01	10	2.1
samp4_DMSO	55.7	2.25	0.28	116	107.68	10	2
samp4_calmidazolium	49.1	2.23	0.44	76.8	43.04	10	1.8
samp4_fludrocortisone	91.9	2.07	1.36	80.4	91.45	10	2
samp5_DMSO	76.1	2.06	1.57	106	81.68	10	2.1
samp5_calmidazolium	35.7	2	1.26	106	37.25	10	2.3
samp5_fludrocortisone	72.9	2.05	0.7	93	96.87	10	2.3
samp6_DMSO	63.5	2.03	0.74	98.8	78.17	10	2
samp6_calmidazolium	22.6	2.17	0.35	32.8	20.45	10	2.2
samp6_fludrocortisone	67.1	2.08	0.32	86.4	74.95	10	2

Supplementary Table S2: Percentage of sequenced reads aligned to the reference genome.
The tables show the overall alignment rate (%) from HISAT2 v2.1.0 for each library prep methods.

Sample name	In-lysate (%)	Purified RNA (%)	TruSeq (%)
samp_1_DMSO	74.16	80.6	68.13
samp_2_DMSO	71.22	75.27	71.78
samp_3_DMSO	74.1	79.22	72.29
samp_4_DMSO	74.2	78.28	68.78
samp_5_DMSO	75.46	78.64	71.82
samp_6_DMSO	75.46	79.59	72.88
samp_1_calmidazolium	74.97	83.22	70.07
samp_2_calmidazolium	73.08	80.24	71.59
samp_3_calmidazolium	76.71	81.64	73.95
samp_4_calmidazolium	74.75	83.73	71.89
samp_5_calmidazolium	75.36	83.92	72.59
samp_6_calmidazolium	72.04	83.54	67.27
samp_1_fludrocortisone	72.82	82.73	70.92
samp_2_fludrocortisone	73.48	81.12	71.74
samp_3_fludrocortisone	76.31	67.24	73.11
samp_4_fludrocortisone	73.61	83.31	70.5
samp_5_fludrocortisone	76.9	82.27	73.11
samp_6_fludrocortisone	73.87	78.07	73.79

Supplementary Table S4: DEGS identified for each method and comparisons. FDR cutoff:0.01. Genes with N/A for log2FC and p-values are labelled as filtered. Data shown in MA plot (Figure 4a)

Method	Comparison	DEG	Non-DEG	filtered
In lysate	Calmidazolium vs DMSO	1197	8850	28476
	Fludrocortisone vs DMSO	688	11243	26592
Purified RNA	Calmidazolium vs DMSO	2822	8587	27114
	Fludrocortisone vs DMSO	1608	12356	24559
TruSeq	Calmidazolium vs DMSO	3210	9552	25761
	Fludrocortisone vs DMSO	2119	12479	23925

Supplementary Table S5: Significant genes identified for each method and comparison. We used FDR cutoff of 0.01 and abs(log2FC)>1. Genes with N/A for log2FC and p-values are labelled as filtered. Significant genes(sig) are further divided into upregulated(up) and down regulated genes. Data shown in Volcano plot (Figure 4b).

Method	Comparison	sig		non-sig	filtered
		up	down		
In lysate	Calmidazolium vs DMSO	195	13	9839	28476
	Fludrocortisone vs DMSO	124	49	11758	26592
Purified RNA	Calmidazolium vs DMSO	239	56	11114	27114
	Fludrocortisone vs DMSO	265	149	13550	24559
TruSeq	Calmidazolium vs DMSO	293	107	12362	25761
	Fludrocortisone vs DMSO	328	217	14053	23925

Supplementary Table S6: Reagent and Time Costs

	In lysate + Smart-3SEQ	Purified + Truseq
Lysis/Extraction	Lysis buffer: ¢35	Qiagen RNeasy: $7
Library Prep	Smart-3SEQ reagents: $5	Illumina Truseq: $152
Total Reagent Cost	$5.35	$159
Total Time	4 hours	2.5 days

Supplementary Table S7: Software and packages versions with non-default parameters and sessionInfo() data.

Software/ packages	Version	parameters
FastQC	v0.11.9	default
MultiQC	1.8	default
Umi_homopolymer.py script	Smart-3SEQ protocol v1.9	UMI sequence, G-overhang extraction, and A-tail removal was done with the umi_homopolymer.py script provided with the Smart-3SEQ protocol v1.9
HISAT2	2.1.0	truseq: hisat2 -x <ht2-index> {-1 <m1> -2 <m2>} -S <sam> , Smart3seq: hisat2 -x <ht2-index> -U <*trim_r> -S <sam>
featureCounts	subread package- 1.6.4	truseq: featureCounts -p -a <annotation_file> -F GTF -t exon -g gene_id -o <output_file> , Smart3seq: featureCounts -a <annotation_file> -F GTF -t exon -g gene_id -s 1 --read2pos 5 -o <output_file>
samtools	1.3.1	default
R	4.0.5	N/A
DESeq2	1.30.1	design: donor + treatment
fgsea	1.16.0	Pathways: hallmark gene sets pathway from MSigDB v7.4

```
R version 4.0.5 (2021-03-31)
Platform: x86_64-apple-darwin17.0 (64-bit)
Running under: macOS Big Sur 10.16
Matrix products: default
LAPACK: /Library/Frameworks/R.framework/Versions/4.0/Resources/lib/libRlapack.dylib

locale:
[1] en_US.UTF-8/en_US.UTF-8/en_US.UTF-8/C/en_US.UTF-8/en_US.UTF-8
attached base packages:
[1] parallel  stats4    stats     graphics  grDevices utils     datasets  methods   base

other attached packages:
 [1] extrafont_0.17              RColorBrewer_1.1-2          data.table_1.14.0           fgsea_1.16.0
 [5] tidyr_1.1.3                 dplyr_1.0.5                 tibble_3.1.1                stringr_1.4.0
 [9] purrr_0.3.4                 readr_1.4.0                 ggpmisc_0.3.9               ggplot2_3.3.3
[13] ROCR_1.0-11                 extraDistr_1.9.1            gridExtra_2.3               cowplot_1.1.1
[17] pheatmap_1.0.12             irr_0.84.1                  lpSolve_5.6.15              gplots_3.1.1
[21] DESeq2_1.30.1               SummarizedExperiment_1.20.0 Biobase_2.50.0              MatrixGenerics_1.2.1
[25] matrixStats_0.58.0          GenomicRanges_1.42.0        GenomeInfoDb_1.26.7         IRanges_2.24.1
[29] S4Vectors_0.28.1            BiocGenerics_0.36.1         MASS_7.3-53.1
loaded via a namespace (and not attached):
 [1] nlme_3.1-152           bitops_1.0-7           bit64_4.0.5            httr_1.4.2             tools_4.0.5
 [6] utf8_1.2.1             R6_2.5.0               KernSmooth_2.23-18     DBI_1.1.1              mgcv_1.8-34
[11] colorspace_2.0-0       withr_2.4.2            tidyselect_1.1.0       extrafontdb_1.0        bit_4.0.4
[16] compiler_4.0.5         cli_2.5.0              DelayedArray_0.16.3    labeling_0.4.2         caTools_1.18.2
[21] scales_1.1.1           genefilter_1.72.1      digest_0.6.27          XVector_0.30.0         pkgconfig_2.0.3
[26] fastmap_1.1.0          rlang_0.4.10           RSQLite_2.2.7          generics_0.1.0         farver_2.1.0
[31] BiocParallel_1.24.1    gtools_3.8.2           RCurl_1.98-1.3         magrittr_2.0.1         polynom_1.4-0
[36] GenomeInfoDbData_1.2.4 Matrix_1.3-2           Rcpp_1.0.6             munsell_0.5.0          fansi_0.4.2
[41] lifecycle_1.0.0        stringi_1.5.3          zlibbioc_1.36.0        grid_4.0.5             blob_1.2.1
[46] crayon_1.4.1           lattice_0.20-41        splines_4.0.5          annotate_1.68.0        hms_1.0.0
[51] locfit_1.5-9.4         knitr_1.33             pillar_1.6.0           geneplotter_1.68.0     fastmatch_1.1-0
[56] XML_3.99-0.6           glue_1.4.2             BiocManager_1.30.12    vctrs_0.3.7            Rttf2pt1_1.3.8
[61] gtable_0.3.0           cachem_1.0.4           xfun_0.22              xtable_1.8-4           survival_3.2-10
[66] AnnotationDbi_1.52.0   memoise_2.0.0          ellipsis_0.3.1
```

Supplementary Figure S1: Bioanalyzer traces of sequencing-ready libraries

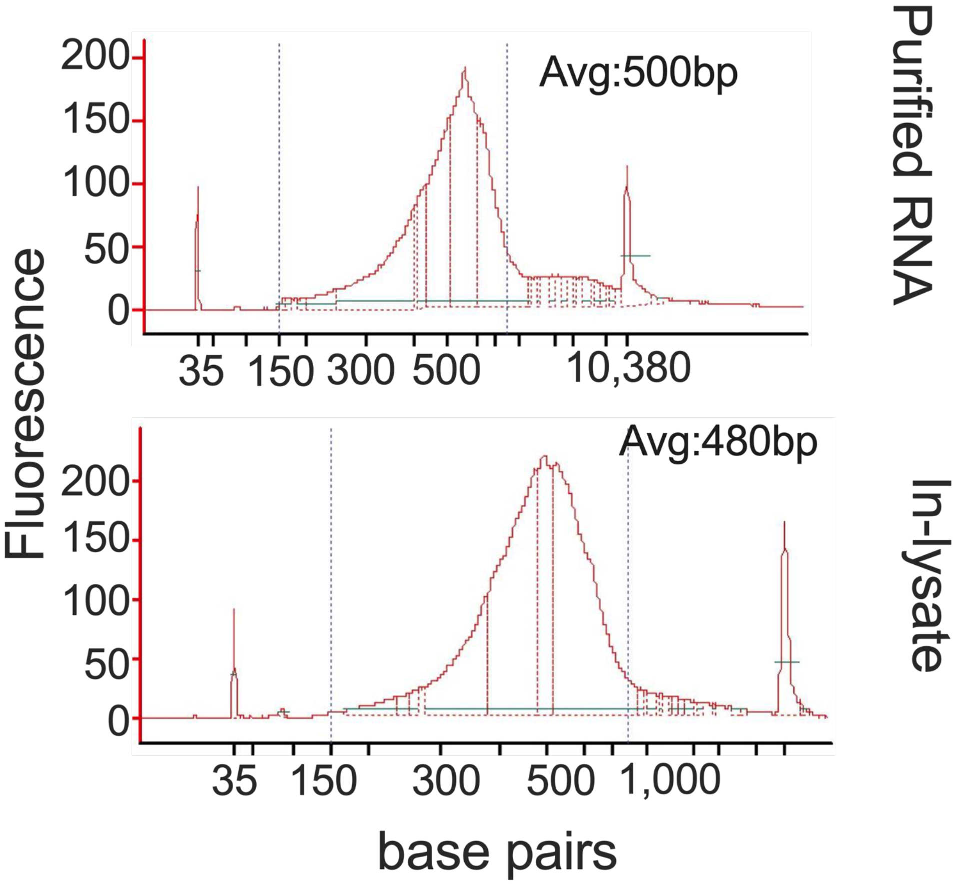

Supplementary Figure S2: Total gene counts in comparison groups. Each dot represents a donor. The y axis is the total uniquely aligned reads in million(M).

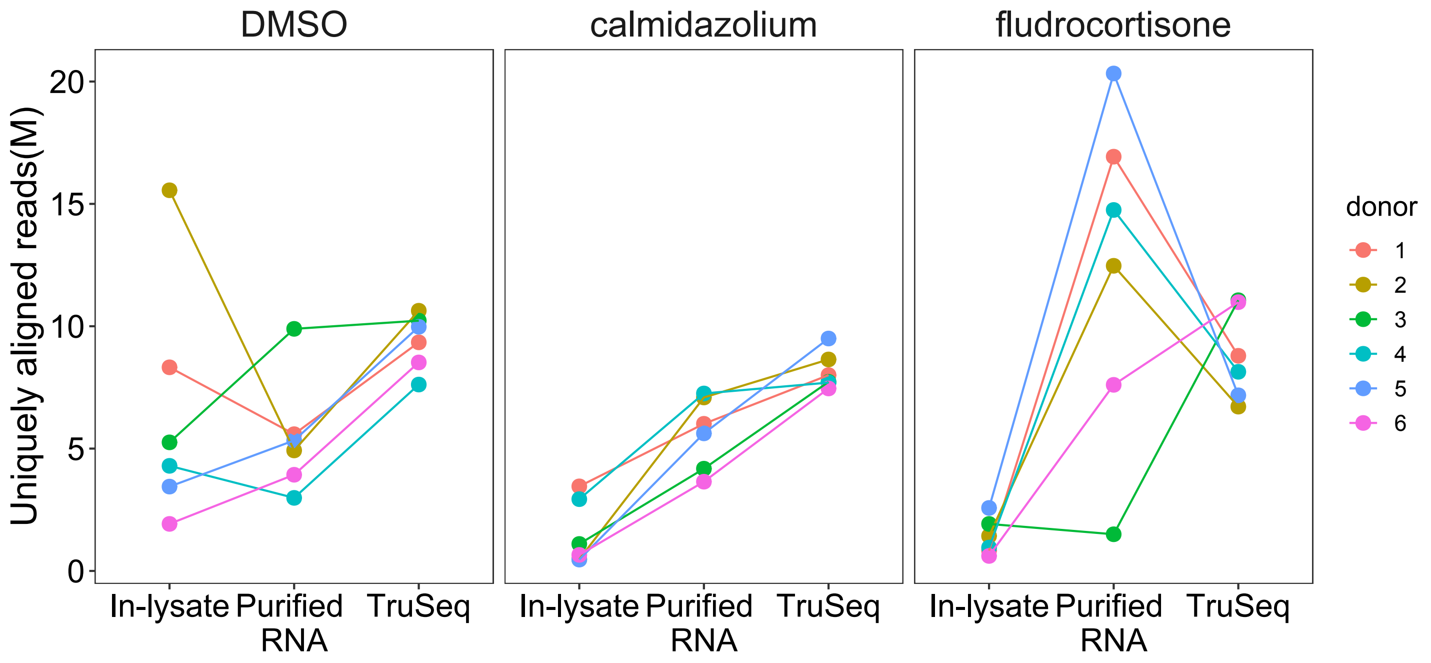

Supplementary Figure S3: uniquely aligned gene reads Correlation including TruSeq. Values in each plot are R^2, Pearson's correlation. Log2(Counts per million) was used to calculate the correlation.

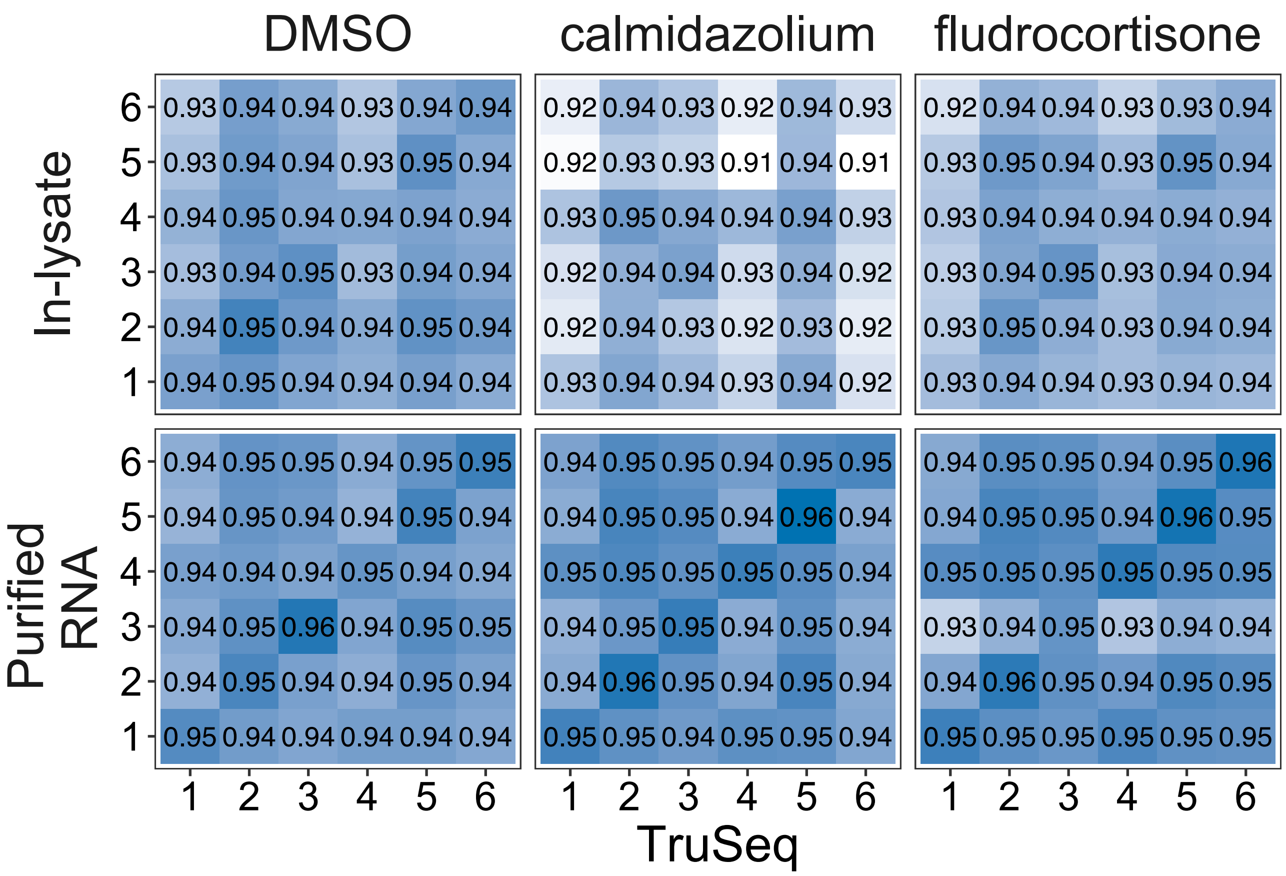

Supplement: Supplementary file 1 — Supplementary Information 1. [file 41598_2021_98912_MOESM1_ESM.pdf]
